# Chronic disease patients during the armed conflict in Sudan: a cross-sectional study on mental health and quality of life

**DOI:** 10.1186/s13031-025-00695-9

**Published:** 2025-07-25

**Authors:** Sohaib Mohammed Mokhtar Ahmed, Moram Elfadel Abdelrhaman Gasmalha, Ahmed Balla M. Ahmed, Khalid Abusofyan Eljezoli Mohammed, Salma Alrawa, Hebatallah Fadhl, Mohanned Abdalsalam Mohammed Salih, Israa Alamin Mohammed Hussein, Asma Mohammed Ahmed Mohammed Salih, Haifa Ibnomer Abdelrahman Elsharif, Hiba Atif Ahmad Elhussein, Sajda Abdelaziz Yahya Abaker, Tagwa Adil Sedahmed Khalefa, Ekhlass Hassan Hdab Hammed, Muhannad Bushra Masaad Ahmed

**Affiliations:** 1 Faculty of Medicine and Health Sciences, University of Gadarif, Gadarif, Sudan; 2 Faulty of Medicine and Health Sciences, Omdurman Islamic University, Omdurman, Sudan; 3 Faculty of Medicine, University of Khartoum, Al-Qasr Street, PO Box: 102, Khartoum, 11111 Sudan; 4 Faculty of Dentistry, University of Gezira, Wad Madani, Sudan; 5 Faculty of Medicine, Aden University, Aden, Yemen; 6 Faculty of Pharmacy, University of Gadarif, Gadarif, Sudan; 7 Faculty of Medicine and Health Sciences, Red Sea University, Port Sudan, Sudan; 8 Gezira College of Medical Sciences and Technology, Wad Madani, Sudan; 9 College of Medicine, Sudan University of Science and Technology, Khartoum, Sudan; 10 Faculty of Medicine, University of Gezira, Wad Madani, Sudan; 11 Faculty of Medicine and Health Sciences, University of Kassala, Kassala, Sudan

**Keywords:** Chronic diseases, Depression, Anxiety, Health-related quality of life, Armed conflict, Sudan

## Abstract

**Background:**

Chronic diseases represent a major global health burden, with their impact becoming even more pronounced in conflict settings. In such environments, the mental health and quality of life of chronic disease patients often deteriorate due to the disruptions caused by war. This study aimed to assess mental health issues among chronic disease patients and evaluate their quality of life during the ongoing armed conflict in Sudan.

**Methods:**

A cross-sectional study was conducted among chronic disease patients in the safest states of Sudan during the current war. Mental health was assessed with the Patient Health Questionnaire-9 for depression and Generalized Anxiety Disorder-7 for anxiety, and quality of life was evaluated using the EuroQol 5-Dimension 5-Level scale. Chi-square tests and Spearman’s rank examined associations between socio-demographic factors and mental health outcomes. Multiple linear regression identified predictors influencing mental health issues, with statistical significance set at *p* ≤ 0.05.

**Results:**

Among 1116 chronic disease patients, the median depression score was 7 (IQR = 8), with 50.3% reporting mild to moderate depression, while the median anxiety score was 6 (IQR = 8), with 47.1% experiencing mild to moderate anxiety. Depression showed significantly strong positive correlation with anxiety (ρ = 0.810, *p* < 0.00). In terms of health-related quality of life, the pain/discomfort domain showed 40.7% of patients reporting moderate to extreme problems, followed by the anxiety/depression domain at 43.4%, making these the most affected areas.

**Conclusion:**

This study revealed high levels of depression and anxiety among Sudanese chronic disease patients during the war, alongside a notably poor quality of life. The findings underscore the urgent need for intervention to implement targeted mental health support programs, aiming to improve the quality of life for this vulnerable population.

## Background

Chronic diseases are defined as conditions that last one year or more and require ongoing medical attention, limit activities of daily living, or both [1]. They pose a significant global health challenge. Conditions such as heart disease, cancer, and diabetes are among the most prevalent, collectively accounting for an estimated 38 million deaths annually, according to the World Health Organization (WHO) [2]. Managing chronic diseases is vital not only to prevent complications and premature mortality but also to maintain a satisfactory quality of life (QoL) [3]. However, the burden of chronic diseases extends far beyond the physical domain, profoundly affecting emotional and mental well-being, which together shape overall health-related quality of life (HRQoL) [4].

These challenges become even more severe in conflict zones [5–7], where healthcare systems often face severe disruptions or complete collapse, significantly limiting access to essential treatments and medications. According to WHO, conflict situations not only exacerbate the spread of preventable diseases but also worsen health outcomes for those already living with chronic conditions [8]. Damaged infrastructure, shortages of medical supplies, and frequent interruptions in treatment increase the risks associated with chronic illnesses [7, 9]. Additionally, the psychological toll of conflict, including heightened stress and trauma, can aggravate conditions such as hypertension and diabetes [10, 11]. Displacement further compounds these issues, as individuals are often cut off from their regular healthcare providers making disease management exceedingly difficult [12, 13].

Comparative studies of affected populations, such as the Rohingya refugees, underscore the profound impact of chronic diseases on overall well-being. For instance, individuals with conditions like cancer report significantly lower scores in physical health domains. Even those without chronic illnesses report poor QoL due to adverse living conditions, including overcrowding, limited access to clean water, and inadequate sanitation [14]. These findings emphasize the need to address both medical needs and broader sociodemographic factors—such as age, employment status, and living conditions—when developing health interventions for displaced communities.

The situation in Sudan exemplifies these issues acutely. Since the onset of conflict in April 2023, Sudan has faced one of the world’s most severe humanitarian crises, displacing over 6.8 million people within the country and forcing 1.5 million to seek refuge in neighboring countries. Nearly half the population—around 24.8 million people—now relies on humanitarian aid for survival [15]. This crisis has placed immense strain on the healthcare system, with approximately 70% of healthcare facilities in conflict-affected regions becoming non-operational and over 30% of public hospitals ceasing to function within the first year of the war [16]. This healthcare collapse has left millions without access to essential medical services [17], disproportionately affecting those with chronic diseases who rely on continuous care and treatment [18].

Despite the severity of these challenges, there was a notable lack of research specifically addressing the experiences of chronic disease patients during the current armed conflict in Sudan, particularly in relation to their mental health and quality of life. This study aimed to assess the mental health issues and quality of life of chronic disease patients during the ongoing conflict in Sudan. By doing so, the study provided insights into the complex challenges faced by this vulnerable population and offered valuable information to guide future interventions aimed at improving their health outcomes in times of crisis.

## Methods

### Study design

This cross-sectional study assessed mental health issues and quality of life among patients with chronic diseases, such as diabetes, hypertension, and chronic kidney disease, during the ongoing war in Sudan. The findings and methodology of this study were meticulously reported in the manuscript in accordance with the STROBE (Strengthening the Reporting of Observational Studies in Epidemiology) guidelines [19].

### Study setting and duration

The research was carried out in both community and hospital settings. The study focused on homes and healthcare facilities in the safest states of Sudan, including Red Sea State, Kassala State, Gadarif State, Northern State, River Nile State, and White Nile State. In hospital settings, participants were recruited from specialized chronic disease centers or hospitals such as diabetes centers, renal centers and hospitals, oncology centers and hospitals, and from specific inpatient wards like those for tuberculosis and asthma, as well as from general outpatient and inpatient departments. In community settings, data collectors identified individuals with chronic diseases through their personal networks—approaching relatives, neighbors, and acquaintances known to be living with chronic conditions. Participants were also referred by others within the community, including those recruited from temporary accommodations, shelters, and internally displaced or refugee camps, allowing for wider reach despite the lack of official patient records due to the ongoing conflict. By gathering data in both participants’ homes and healthcare facilities, the study captured a more comprehensive understanding of their experiences within their local communities and formal healthcare environments.

Data collection took place from September 16 to December 2, 2024.

### Participants and sampling

The target population comprised individuals aged 18 years or older diagnosed with any chronic disease, including diabetes, hypertension, cardiovascular diseases, respiratory diseases, and other long-term conditions. Only those who had been living with these conditions for at least one year were included, to capture the effect of the disease on their mental health and quality of life. Other inclusion criteria required participants to provide written informed consent and have the cognitive ability to complete the interview.

The sample size was estimated using the Cochrane formula, considering the unavailability of official population records in Sudan. Assuming a population proportion of 50%, a margin of error of 5%, and a confidence interval of 95%, the minimum required sample size was calculated to be 385 participants. We aimed to include as many patients with chronic diseases as possible during the data collection period, ultimately recruiting 1116 individuals.

Convenience sampling was employed in this study due to the challenges posed by the ongoing conflict in Sudan, which made accessing official records of the patients impossible. While this non-probability sampling method limits generalizability, the large sample size helps enhance the diversity of the sample and allows for more precise estimation of outcomes within the study context.

### Data collection procedures

Data collection was carried out by trained medical students who conducted structured face-to-face interviews with participants. The interviews were conducted in a confidential manner to encourage honest and accurate responses while maintaining participants’ privacy. These medical students received training to ensure consistency, accuracy, and adherence to ethical standards during data collection.

### Data collection tools

The data collection tool used in this study is structured into three sections. The first section gathers basic demographic details such as age, sex, marital status, highest educational level, employment status, living situation, internal displacement status, income level (self-reported), and chronic disease diagnoses, with a total of 9 questions. The second section included the Arabic versions of PHQ-9 (Patient Health Questionnaire-9) and GAD-7 (Generalized Anxiety Disorder-7) scales [20], which together measure the severity of depressive and anxiety symptoms over the past two weeks, with 9 and 7 questions, respectively. According to PHQ-9 scoring guidelines, scores of 0–4 indicate no or minimal depression, 5–9 mild, 10–14 moderate, 15–19 moderately severe, and 20–27 severe depression [21].

For GAD-7, scores of 0–4 indicate minimal anxiety, 5–9 mild, 10–14 moderate, and ≥ 15 severe anxiety [22]. The final section utilized the Arabic version of EQ-5D-5 L (EuroQol 5-Dimension 5-Level) scale for Quality of Life Assessment [23], consists of 5 questions that evaluate mobility, self-care, usual activities, pain/discomfort, and anxiety/depression, each on a 5-point scale. Each dimension is rated on a 5-point scale: 1 = no problems, 2 = slight problems, 3 = moderate problems, 4 = severe problems, and 5 = extreme problems or inability. Responses across the five dimensions generate a 5-digit health state profile (e.g., 21111), with higher numbers indicating greater impairment [24]. To minimize selection and reporting bias, data were collected using a standardized online questionnaire with all fields marked as mandatory, thereby eliminating missing responses. Additionally, data collection was anonymous, which helped reduce social desirability bias and encouraged honest reporting.

### Data analysis

Descriptive and inferential statistical analyses were performed using SPSS version 27. Continuous variables, such as age and mental health scores, were summarized using medians and interquartile ranges due to non-normal distributions, while categorical variables were presented as frequencies and percentages. The primary outcomes were depression and anxiety scores, measured using the PHQ-9 and GAD-7 scales, respectively, both treated as continuous variables. Predictor variables included sex, marital status, highest educational level, employment status, internal displacement, income level, and living situation. Potential confounders—defined as variables that could influence both the predictors and outcomes—were age and comorbidity burden, and were adjusted using multiple linear regression analysis.

Bivariate analyses were conducted to assess associations between socio-demographic factors and mental health outcomes. Specifically, chi-square (χ²) tests were used to compare categorical variables (e.g., sex, income level, living situation) with categorized depression and anxiety levels, while Spearman’s rank correlation coefficient was used to examine the relationship between depression and anxiety scores.

Multiple linear regression analyses were then performed to identify independent predictors of depression and anxiety, adjusting for potential confounders. Reference categories were chosen based on clinical relevance or the lowest logical category (e.g., females for sex, married for marital status, illiterate for education). Predictor inclusion was based on theoretical relevance, supporting literature, and biological plausibility, irrespective of univariate statistical significance. Statistical significance was defined as *p* ≤  0.05. Regression results were reported as unstandardized coefficients, standard errors, and associated p-values.

## Results

### Socio-demographic characteristics of participants

A total of 1116 participants were included in the study, with a median age of 44 years (IQR = 29). Females constituted the majority (60.1%), and 54.5% of participants were married. Educational levels varied, with 48.8% holding a bachelor’s degree and 22.4% completing high school. Employment status was diverse with 38.7% were unemployed and 23.0% were students. In terms of living situation, 48.6% lived with family or friends while 22.6% in rented houses and 2.3% in Internally displaced/refugee camps. Internally displaced individuals accounted for 58.3% of participants. Income levels were reported as average by 54.7% (Table 1).

**Table 1:** Socio-demographic characteristics of participants (*N* = 1116)

Socio-demographic factors	Overall (*N* = 1116)	Socio-demographic factors	Overall (*N* = 1116)
**Age**		**Employment status**	
Median (IQR)	44 (29)	Student	256 (23.0%)
**Sex**		Unemployed	432 (38.7%)
Female	671 (60.1%)	Self-employed	164 (14.7%)
Male	445 (39.9%)	Government employee	171 (15.3%)
**Marital status**		Private sector employee	92 (8.3%)
Married	608 (54.5%)	**Living situation**	
Single	384 (34.4%)	With family and friends	542 (48.6%)
Divorced	38 (3.4%)	Own house	203 (18.2%)
Widow	86 (7.7%)	Rented house	252 (22.6%)
**Highest educational level**		Temporary accommodation/Shelter	93 (8.3%)
Illiterate	49 (4.4%)	Internally displaced/Refugee camp	26 (2.3%)
Primary school	140 (12.5%)	**Internal displacement**	
High school	250 (22.4%)	No	465 (41.7%)
bachelor’s degree	545 (48.8%)	Yes	650 (58.3%)
Higher studies	72 (6.5%)	**Income level**	
Informal education (Khalwa)	60 (5.4%)	Low	155 (13.9%)
		Below average	201 (18.0%)
		Average	611 (54.7%)
		More than average	126 (11.3%)
		High	23 (2.1%)

### Chronic diseases

**Fig. 1:**
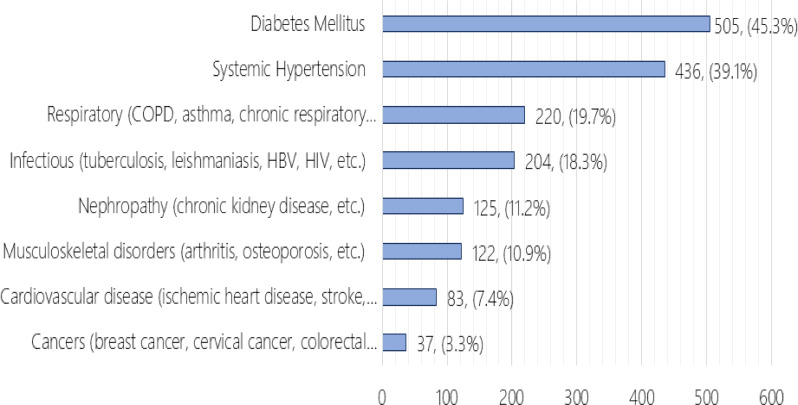
Prevalence of chronic diseases among participants (*N* = 1116). *Note*: Participants were allowed to select more than one chronic disease

The most common chronic condition was diabetes mellitus, affecting 45.3% of participants, followed by systemic hypertension (39.1%). Respiratory diseases (19.7%), infectious diseases such as tuberculosis and hepatitis (18.3%), nephropathy (11.2%), musculoskeletal disorders (10.9%), cardiovascular diseases (7.4%), and cancers (3.3%) were also reported (Fig. 1).

### Mental health

Depression (PHQ-9) had a median score of 7 (IQR = 8), with 33.6% showing none-minimal depression, 30.8% mild, and 5.2% severe depression. Anxiety (GAD-7) had a median score of 6 (IQR = 8), with 44.3% reporting minimal anxiety, 31.2% mild, and 8.6% severe anxiety (Table 2). Depression showed significantly strong positive correlation with anxiety (ρ = 0.810, *p* < 0.00).

**Table 2:** Scores and categorization for depression and anxiety among participants (*N* = 1116)

Variables	*N* (1116)	Variables	*N* (1116)
**Depression** (PHQ-9)		**Depression**	
Median (IQR)	7 (8)	None-Minimal	375 (33.6%)
**Anxiety** (GAD-7)		Mild	344 (30.8%)
Median (IQR)	6 (8)	Moderate	218 (19.5%)
		Moderately Severe	121 (10.8%)
		Severe	58 (5.2%)
		**Anxiety**	
		Minimal Anxiety	494 (44.3%)
		Mild Anxiety	348 (31.2%)
		Moderate Anxiety	178 (15.9%)
		Severe Anxiety	96 (8.6%)

Mental health analyses revealed significant associations for depression and anxiety with sex, income, and living situation. Females reported higher scores for both depression (χ² = 9.97, *p* = 0.002) and anxiety (χ² = 8.44, *p* = 0.004). Lower income correlated with worse mental health, as participants with low income had the highest depression (χ² = 74.6, *p* < 0.001) and anxiety scores (χ² = 62.3, *p* < 0.001). Temporary accommodations were linked to higher depression (*p* < 0.001) and anxiety scores (*p* < 0.001) compared to stable housing.

Table 3 shows the multiple linear regression analysis of mental health predictors. For sex, males had lower scores for both depression (*p* = 0.007) and anxiety (*p* = 0.002) compared to females. Regarding marital status, widowed individuals had significantly higher depression scores (*p* = 0.018). In terms of education, high school graduates showed increased depression (*p* = 0.05) and anxiety (*p* = 0.045) compared to illiterate individuals. Employment status was a strong predictor, with unemployed and employed individuals in both government and private sectors reporting significantly lower depression and anxiety scores (all *p* < 0.001), compared to students. Similarly, higher income levels were associated with lower depression and anxiety scores, with significant reductions observed across all income categories above “low” (all *p* < 0.001). For living situations, residing in temporary accommodations was linked to higher depression scores (*p* = 0.038).

**Table 3:** Predictors of depression and anxiety among the study participants (*N* = 1116)

Predictor	Depression			Anxiety		
	Estimate	SE	*p*	Estimate	SE	*p*
**Sex:**						
Male – Female	-1.104	0.4052	0.007	-1.1047	0.36036	0.002
**Marital status**: (Reference: Married)						
Single	0.877	0.58359	0.133	-0.02822	0.51901	0.957
Divorced	-0.773	0.99699	0.438	-0.08669	0.88666	0.922
Widow	1.701	0.71675	0.018	1.19828	0.63743	0.06
**Highest educational level achieved**: (Reference: illiterate)						
Primary school	0.844	1.00234	0.4	0.50086	0.89141	0.574
High school	1.888	0.96356	0.05	1.72179	0.85693	0.045
bachelor’s degree	1.455	0.96729	0.133	1.55257	0.86024	0.071
Higher studies	1.264	1.17477	0.282	1.14819	1.04476	0.272
Informal education (Khalwa)	2.249	1.15482	0.052	1.38479	1.02702	0.178
**Employment status**: (Reference: Student)						
unemployed	-2.549	0.64998	< 0.001	-2.04519	0.57805	< 0.001
Self-employed	-2.577	0.78503	0.001	-1.19793	0.69815	0.086
Government employee	-2.577	0.75784	< 0.001	-1.79773	0.67398	0.008
Private sector employee	-3.523	0.90656	< 0.001	-3.02393	0.80623	< 0.001
**Living situation**: (Reference: With family and friends)						
Own house	-0.329	0.55278	0.552	-0.5965	0.49161	0.225
Rented house	0.398	0.47773	0.405	-0.31662	0.42486	0.456
Temporary accommodation/Shelter	1.43	0.68883	0.038	0.81126	0.6126	0.186
IDP/Refugee camp	-1.338	1.23174	0.278	-1.42384	1.09543	0.194
**Internal displacement**:						
Yes – No	0.646	0.39876	0.106	0.46055	0.35463	0.194
**Income level**: (Reference: Low)						
Below average	-2.386	0.65398	< 0.001	-1.92862	0.58161	< 0.001
Average	-3.114	0.57903	< 0.001	-2.92327	0.51495	< 0.001
More than average	-2.592	0.76541	< 0.001	-2.916	0.6807	< 0.001
High	-5.349	1.36555	< 0.001	-4.29309	1.21443	< 0.001

### Health-related quality of life

Health-related quality of life (HRQoL) was assessed using the EQ-5D-5 L scale. Mobility issues were absent in 62.1% of participants, while 18.9% reported slight problems, and 9.9% faced moderate issues. Severe and extreme mobility impairments were noted in 6.8% and 2.2%, respectively. Pain/discomfort was prevalent, with only 37.4% reporting no issues, while moderate and severe pain were reported by 25.2% and 12.1%, respectively. Anxiety/depression levels varied, with 35.0% experiencing no anxiety/depression, while moderate, severe, and extreme levels were reported by 25.0%, 13.6%, and 4.8%, respectively (Table 4).

**Table 4:** Health-related quality of life (HRQoL) dimensions and severity levels of participants using EQ-5D-5 L scale (*N* = 1116)

Severity level/dimensions	Mobility	Self-care	Usual activity	Pain/discomfort	Anxiety/depression
**Level 1** (No problems)	693 (62.1%)	795 (71.2%)	666 (59.7%)	417 (37.4%)	390 (35.0%)
**Level 2** (Slight problems)	211 (18.9%)	147 (13.2%)	165 (14.8%)	245 (22.0%)	241 (21.6%)
**Level 3** (Moderate problems)	111 (9.9%)	89 (8.0%)	169 (15.1%)	281 (25.2%)	279 (25.0%)
**Level 4** (Severe problems)	76 (6.8%)	54 (4.8%)	74 (6.6%)	135 (12.1%)	152 (13.6%)
**Level 5** (Extreme problems/ unable to do)	25 (2.2%)	31 (2.8%)	42 (3.8%)	38 (3.4%)	53 (4.8%)

## Discussion

This study is one of its kind among conflict-related research. It focuses on studying the mental health issues and quality of life of chronically ill patients, a particularly affected and vulnerable population during the ongoing conflict in Sudan.

The most prevalent chronic diseases in our sample were diabetes mellitus and hypertension, each affecting nearly half of the participants. While our sample is limited to individuals with chronic diseases, this distribution exceeds their reported prevalence among approximately one-fifth of the general Sudanese population [25], suggesting that our non-probability sample may reflect broader national disease patterns. These findings are also consistent with, and exceed, the high prevalence of hypertension (33.3%) and diabetes (12.2%) reported in North Africa [26].

More than a third of participants had symptoms suggestive of depression and almost a quarter of them had symptoms suggestive of anxiety. Higher levels of both depression and anxiety symptoms were reported early during the war in Sudan [27, 28]. This may be related to humans’ ability to adapt to prolonged stressors [29]. Notably, chronic conditions themselves have a well-documented negative effect on mental health, with pain serving as a potential mediator in this association [30]. Consistent with previous research, our study revealed a strong positive correlation between depression and anxiety, where higher levels of depression were significantly associated with increased anxiety [31]. This pattern is not limited to individuals with chronic diseases but is a well-established trend in the general population. For example, a 2022 study among university students during the COVID-19 pandemic also reported a strong positive association between depression and anxiety symptoms [32].

When examining the predictors of depression and anxiety, male participants had lower scores for both conditions compared to females. This aligns with well-established trends among chronic renal failure, where women consistently report higher levels of depression and anxiety than men [33]. This difference may be partly attributed to sociocultural roles and expectations, as women—particularly in traditional societies—often bear greater emotional, caregiving, and household responsibilities, which may increase psychological distress. Additionally, men may underreport emotional symptoms due to cultural norms discouraging emotional expression, potentially contributing to lower observed scores. Regarding marital status, widowed individuals exhibited significantly higher depression scores. This finding is consistent with studies from the United States, Europe, Korea, and China, which show that depression tends to peak within the first year of widowhood for both men and women, although women often recover to levels comparable to their married peers over time [34]. The elevated depression levels among widowed individuals may stem from the profound emotional loss, social isolation, and disruption of daily routines that often follow the death of a spouse, particularly in cultures where marriage plays a central role in emotional and social support. High school graduates in our study reported higher depression and anxiety levels compared to illiterate individuals. This finding contrasts with a previous study among hospitalized COVID-19 patients, which found a negative association between educational status and psychological distress [31]. This discrepancy may be explained by differences in study populations and disease types. Our participants were living with chronic illnesses, which often require long-term self-management and carry ongoing health, financial, and social burdens. Individuals with higher education may have greater awareness of their illness trajectory and complications, leading to increased worry and emotional distress. In contrast, acute illnesses like COVID-19 may generate immediate fear but with more optimism for recovery, and higher education in that context might help with coping and health literacy.

Unemployed and employed individuals in both the government and private sectors reported significantly lower depression and anxiety scores compared to students in our study. However, this contrasts with well-established evidence showing that unemployment is positively associated with mental health disorders, particularly anxiety and depression [35], while employment often serves as a protective factor by providing financial stability, routine, and a sense of purpose [36]. The unexpected finding regarding unemployment may be due to a type II error, as unemployed individuals made up more than one-third of our sample, potentially diluting detectable differences due to sample imbalance. Residing in temporary accommodations was associated with higher depression scores in our study. This aligns with existing evidence showing that unsheltered populations face disproportionately high rates of chronic illnesses and serious mental health conditions [37], likely due to instability, poor living conditions, and limited access to healthcare and social support. Our study found that higher income levels were associated with lower depression and anxiety scores. This is supported by recent research showing that individuals with the lowest incomes are significantly more likely to experience anxiety and depressive disorders, highlighting the strong link between poverty and poor mental health [38]. Comorbid anxiety and depression can negatively impact medication adherence, compromise treatment effectiveness and safety, and contribute to the development and progression of non-communicable diseases, ultimately increasing mortality from these conditions [39].

Regarding health related quality of life, forty or more percent of patients had moderate to extreme problems in pain/discomfort and anxiety/depression domains. Pain/discomfort and Anxiety/depression are the most affected domains with chronic diseases in a wide range of studies [40–42]. Chronic pain is a frequent symptom of many chronic diseases that impairs the quality of life [43]. Similarly anxiety is common among individuals with chronic diseases and negatively impacts their quality of life [44, 45].

This study provides valuable insights into mental health issues and quality of life among individuals with chronic diseases during the war in Sudan. The main limitations include the cross-sectional design, which does not allow for the establishment of causality, and the use of a non-probability sampling method, which limits the generalizability of the findings and may introduce selection bias that cannot be corrected by increasing the sample size. The use of self-reported data may be subject to recall bias and social desirability bias, potentially affecting the accuracy of responses. Additionally, while patients with multimorbidity were included, reporting conditions separately may have masked the overall burden of multiple illnesses and their impact on mental health. Despite these limitations, the study included a large and diverse sample of individuals with chronic conditions across multiple states in Sudan, providing meaningful evidence from a highly vulnerable population during an active conflict.

## Conclusion

There was a high level of depression and anxiety among Sudanese individuals with chronic diseases during the war, along with a poor quality of life. Anxiety/depression and pain/discomfort were the most affected domains of quality of life. Depression showed a significantly strong positive correlation with anxiety. These findings can guide humanitarian and healthcare policymakers in prioritizing mental health support and integrating psychosocial care into chronic disease management during conflicts. Addressing both mental and physical health needs is essential in ensuring holistic care for vulnerable populations in conflict-affected settings. Future studies should address the limitations of this research, and qualitative studies are needed to further explore the observed associations.

## Data Availability

The datasets used and/or analysed during the current study are available from the corresponding author on reasonable request.

## Abbreviations

WHO: World Health Organization
QoL: Quality of Life
HRQoL: Health-Related Quality of Life
STROBE: Strengthening the Reporting of Observational Studies in Epidemiology
PHQ-9: Patient Health Questionnaire-9
GAD-7: Generalized Anxiety Disorder-7
EQ-5D-5L: EuroQol 5-Dimension 5-Level

## References

[1] About Chronic Diseases [Internet]. Centers for Disease Control and Prevention. 2022. Available from: https://www.cdc.gov/chronicdisease/about/index.htm

[2] World Health Organization. Noncommunicable Diseases [Internet]. www.who.int.2020. Available from: https://www.who.int/health-topics/noncommunicable-diseases#tab=tab_1

[3] Tóthová V, Bártlová S, Dolák F, Kaas J, Kimmer D, Maňhalová J et al. Quality of life in patients with chronic diseases. Neuro Endocrinology Letters [Internet]. 2014;35 Suppl 1:11–8. Available from: https://pubmed.ncbi.nlm.nih.gov/25433349/25433349

[4] Megari K. Quality of life in chronic disease patients. Health Psychol Res. 2013;1(3):27. 10.4081/hpr.2013.e27 PMC4768563 26973912

[5] Piccoli GB, Brunori G, Gesualdo L, Kalantar-Zadeh K. The impact of the Russian–Ukrainian war for people with chronic diseases. Nat Rev Nephrol. 2022;18(7):411–2. 35444237 10.1038/s41581-022-00574-z

[6] Zeleke TK, Ayal BM, Chanie GS, Alemu MA. Liknaw Workie Limenh, Malede Berihun Yismaw, Worsening of Medication Non-adherence among Patients with Chronic Diseases During Times of Armed Conflict in the War-Torn Region of Ethiopia. Scientific African [Internet]. 2024;25(2468– 2276):e02336. Available from: https://www.sciencedirect.com/science/article/pii/S2468227624002795

[7] Tesfay Gebrehiwet HT, Abebe, Woldemichael A, Kibrom B, Gebresilassie M, Tsadik AA, Asgedom, et al. War and health care services utilization for chronic diseases in rural and semiurban areas of tigray, Ethiopia. JAMA Netw Open. 2023;6(8):e2331745. 37651138 10.1001/jamanetworkopen.2023.31745 PMC10472195

[8] Strengthening NCD. integration in humanitarian emergencies [Internet]. www.who.int. Available from: https://www.who.int/activities/strengthening-ncd-integration-in-humanitarian-emergencies

[9] Pedersen D. Political violence, ethnic conflict, and contemporary wars: broad implications for health and social well-being. Social Science & Medicine [Internet]. 2002;55(2):175–90. Available from: https://www.sciencedirect.com/science/article/abs/pii/S0277953601002611 10.1016/s0277-9536(01)00261-1 12144134

[10] Ospelt E, Mungmode A, Hardison H, Wirsch A, Rioles N, Dawson J et al. The Impact of War and Conflict on People Living with Diabetes: A Scoping Review. Clinical Diabetology [Internet]. 2024;13(6):373–85. Available from: https://journals.viamedica.pl/clinical_diabetology/article/view/100617

[11] Shalimova A, Stoenoiu MS, Cubała WJ, Burnier M, Persu A, Narkiewicz K. The impact of war on the development and progression of arterial hypertension and cardiovascular disease: protocol of a prospective study among Ukrainian female refugees. Frontiers in Cardiovascular Medicine [Internet]. 2024 Jan 11 [cited 2024 Feb 15];10:1324367. Available from: https://www.ncbi.nlm.nih.gov/pmc/articles/PMC10808621/10.3389/fcvm.2023.1324367 PMC10808621 38274316

[12] Abu Khudair S, Khader YS, Morrissey H, El-Khatib Z, Sandor J. Factors associated with suboptimal adherence to hypertensive medications among Syrian Refugees – Cross-Sectional study at the Zaatari camp, Jordan. Patient Prefer Adherence. 2021;15:2125–35. 34584406 10.2147/PPA.S327903 PMC8464360

[13] Mesfin B, Alexander Mersha Demise, Shiferaw M, Freweyni Gebreegziabher F, Girmaw. The Effect of Armed Conflict on Treatment Interruption, Its Outcome and Associated Factors Among Chronic Disease Patients in North East, Amhara, Ethiopia, 2022. Patient Related Outcome Measures [Internet]. 2023 Aug 1 [cited 2024 Jan 23];14:243–51. Available from: https://www.ncbi.nlm.nih.gov/pmc/articles/PMC10463179/10.2147/PROM.S388426 PMC10463179 37649898

[14] Hossain A, Baten A, Saadi A, Rana J, Rahman T, Hasan M, Reza et al. Chronic Illness and Quality of Life 5 Years After Displacement Among Rohingya Refugees in Bangladesh. JAMA Network Open [Internet]. 2024 Sep 3 [cited 2024 Sep 18];7(9):e2433809–9. Available from: https://jamanetwork.com/journals/jamanetworkopen/fullarticle/2823606#google_vignette 10.1001/jamanetworkopen.2024.33809 PMC11409150 39287945

[15] Sudan.| Displacement Tracking Matrix [Internet]. dtm.iom.int. International Organization for Migration. Available from: https://dtm.iom.int/sudan

[16] Badri R, Dawood I. The implications of the Sudan war on healthcare workers and facilities: a health system tragedy. Conflict and Health [Internet]. 2024;18(1). Available from: https://conflictandhealth.biomedcentral.com/articles/10.1186/s13031-024-00581-w 10.1186/s13031-024-00581-w PMC10946115 38494471

[17] Khogali A, Homeida A. Impact of the 2023 armed conflict on Sudan’s healthcare system. Public Health Challenges. 2023;2(4). 10.1002/puh2.134 PMC12039653 40496780

[18] World Health Organization (WHO). Sudan health emergency: situation report No. 2, 15 June – 16 July 2023 [Internet]. 2023. Available from: https://extranet.who.int/ssa/Index.aspx

[19] von Elm E, Altman DG, Egger M, Pocock SJ, Gøtzsche PC, Vandenbroucke JP, STROBE Initiative. Strengthening the reporting of observational studies in epidemiology (STROBE) statement: guidelines for reporting observational studies. BMJ. 2007;335(7624):806–8. 10.1136/bmj.39335.541782.AD. PMID: 17947786; PMCID: PMC2034723. 17947786 10.1136/bmj.39335.541782.AD PMC2034723

[20] Sawaya H, Atoui M, Hamadeh A, Zeinoun P, Nahas Z. Adaptation and initial validation of the patient health questionnaire – 9 (PHQ-9) and the generalized anxiety Disorder – 7 questionnaire (GAD-7) in an Arabic speaking Lebanese psychiatric outpatient sample. Psychiatry Res. 2016;239:245–52. 27031595 10.1016/j.psychres.2016.03.030

[21] Patient Health Questionnaire-9 (PHQ-9). - Mental Disorders Screening [Internet]. National HIV Curriculum. 2025. Available from: https://www.hiv.uw.edu/page/mental-health-screening/phq-9

[22] Generalized Anxiety Disorder 7-item (GAD-7) - Mental Disorders Screening [Internet]. National HIV Curriculum. 2024. Available from: https://www.hiv.uw.edu/page/mental-health-screening/gad-7

[23] Bekairy AM, Bustami RT, Almotairi M, Jarab A, Katheri AM, Aldebasi TM et al. Validity and reliability of the Arabic version of the EuroQOL (EQ-5D). A study from Saudi Arabia. International journal of health sciences [Internet]. 2018;12(2):16–20. Available from: https://pubmed.ncbi.nlm.nih.gov/29599689/PMC5870320 29599689

[24] EuroQol Research Foundation. EQ-5D User Guides – EQ-5D [Internet]. 2019. Available from: https://euroqol.org/publications/user-guides

[25] Almobarak AO, Badi S, Siddiq SB, Noor SM, Elmadhoun WM, Suliman M, et al. The prevalence and risk factors for systemic hypertension among Sudanese patients with diabetes mellitus: A survey in diabetes healthcare facility. Diabetes Metabolic Syndrome: Clin Res Reviews. 2020;14(6):1607–11. 10.1016/j.dsx.2020.08.010 32866934

[26] Abdelbagi O, Musa IR, Musa SM, ALtigani SA, Adam I. Prevalence and associated factors of hypertension among adults with diabetes mellitus in Northern sudan: a cross-sectional study. BMC Cardiovasc Disord. 2021;21(1). 10.1186/s12872-021-01983-x PMC8037914 33838664

[27] Sahar MMB, Elmahdi M, Ahmed. Mental health consequences among Sudanese due to the armed conflicts and civil unrest of 2023: a cross-sectional study. Int J Soc Psychiatry. 2024;70(3). 10.1177/00207640231221101 38214246

[28] Balla A, Yeddi AA, Alrawa SS, Esraa SA, Alfadul. Anxiety and depression symptoms among a sample of Khartoum civilians during the 2023 Sudan armed conflict: A cross-sectional study. PLoS ONE. 2024;19(7):e0307648–8. 39052622 10.1371/journal.pone.0307648 PMC11271933

[29] Shuster A, O’Brien M, Luo Y, Berner LA, Perl O, Heflin M et al. Emotional adaptation during a crisis: decline in anxiety and depression after the initial weeks of COVID-19 in the United States. Translational Psychiatry. 2021;11(1). 10.1038/s41398-021-01552-y PMC8377451 34417441

[30] Ma Y, Xiang Q, Yan C, Liao H, Wang J. Relationship between chronic diseases and depression: the mediating effect of pain. BMC Psychiatry. 2021;21(1). 10.1186/s12888-021-03428-3 PMC8419946 34488696

[31] Arzu Kaplanoglu, Sonmez A, Yildirim O, Feride Cogullular E, Yurtsever, Oz B et al. Anxiety and Depression-Related Factors in Hospitalized Patients Diagnosed With Coronavirus Disease 2019: A Detailed Cross-Sectional Analysis From a Tertiary Center. Cureus [Internet]. 2025 Jan 23 [cited 2025 Jul 16];17(1). Available from: https://pmc.ncbi.nlm.nih.gov/articles/PMC11846142/10.7759/cureus.77865 PMC11846142 39991325

[32] Gao D, Xiang Q, Lu G, Tong J, Jiang W, Yu X et al. Evaluation and analysis of anxiety and depression symptoms for college students during COVID-19 pandemic. BMC Psychol. 2022;10(1). 10.1186/s40359-022-00934-1 PMC9523640 36180957

[33] Theofilou P. Depression and anxiety in patients with chronic renal failure: the effect of sociodemographic characteristics. Int J Nephrol. 2011;2011:1–6. 10.4061/2011/514070 PMC3118662 21716702

[34] Jadhav A, Weir D. Widowhood and Depression in a Cross-National Perspective: Evidence from the United States, Europe, Korea, and China. The Journals of Gerontology: Series B [Internet]. 2017;73(8):e143–53. Available from: https://www.ncbi.nlm.nih.gov/pmc/articles/PMC6178968/pdf/gbx021.pdf 10.1093/geronb/gbx021 PMC6178968 28329854

[35] Yang Y, Niu L, Amin S, Yasin I. Unemployment and mental health: a global study of unemployment’s influence on diverse mental disorders. Frontiers in Public Health [Internet]. 2024;12. Available from: https://pmc.ncbi.nlm.nih.gov/articles/PMC11672120/10.3389/fpubh.2024.1440403 PMC11672120 39735766

[36] Evans L, Lund C, Massazza A, Weir H, Fuhr DC. The impact of employment programs on common mental disorders: A systematic review. Int J Soc Psychiatry. 2022;68(7):002076402211046. 10.1177/00207640221104684 PMC9548920 35796434

[37] Richards J, Kuhn R. Unsheltered homelessness and health: A literature review. AJPM Focus [Internet]. 2022;2(1):100043. Available from: https://www.sciencedirect.com/science/article/pii/S2773065422000414 10.1016/j.focus.2022.100043 PMC10546518 37789936

[38] Ridley M, Rao G, Schilbach F, Patel V. Poverty, depression, and anxiety: causal evidence and mechanisms. Science [Internet]. 2020;370(6522). Available from: https://science.sciencemag.org/content/370/6522/eaay0214 10.1126/science.aay0214 33303583

[39] Mehari EA, Kidane RB, Areki MF, Seid AM, Abebech Tewabe Gelaye. The magnitude and associated factors of anxiety and depression among non-communicable chronic disease patients during COVID-19 pandemic in a resource-limited setting. Clin Epidemiol Global Health. 2023;21:101274–4. 10.1016/j.cegh.2023.101274 PMC10028352 37033721

[40] Arifin B, Idrus LR, van Asselt ADI, Purba FD, Perwitasari DA, Thobari JA, et al. Health-related quality of life in Indonesian type 2 diabetes mellitus outpatients measured with the Bahasa version of EQ-5D. Qual Life Res. 2019;28(5):1179–90. 30649698 10.1007/s11136-019-02105-z PMC6470109

[41] Shah S, Abbas G, Aslam A, Fawad Ahmad Randhawa FU, Khan H, Khurram, et al. Assessment of health-related quality of life among patients with obesity, hypertension and type 2 diabetes mellitus and its relationship with multimorbidity. PLoS ONE. 2023;18(8):e0289502–2. 37540689 10.1371/journal.pone.0289502 PMC10403106

[42] Shimels T, Kassu RA, Bogale G, Muleta MB, Akalu GT, Getachew A et al. Health-Related Quality of Life of Patients with Type 2 Diabetes Mellitus and Hypertension in Addis Ababa, Ethiopia. Ethiopian Journal of Health Sciences [Internet]. 2022 Mar 1 [cited 2022 Oct 28];32(2):381–92. Available from: https://pubmed.ncbi.nlm.nih.gov/35693563/10.4314/ejhs.v32i2.19 PMC9175229 35693563

[43] Kawai K, Kawai AT, Wollan P, Yawn BP. Adverse impacts of chronic pain on health-related quality of life, work productivity, depression and anxiety in a community-based study. Fam Pract. 2017;34(6):656–61. 28444208 10.1093/fampra/cmx034 PMC6260800

[44] Härter MC, Conway KP, Merikangas KR. Associations between anxiety disorders and physical illness. Eur Arch Psychiatry Clin NeuroSci. 2003;253(6):313–20. 14714121 10.1007/s00406-003-0449-y

[45] Baghdadi LR, Alhassan MK, Alotaibi FH, AlSelaim KB, Alzahrani AA, AlMusaeed FF. Anxiety, depression, and common chronic diseases, and their association with social determinants in Saudi primary care. J Prim Care Community Health. 2021;12:215013272110549. 10.1177/21501327211054987 PMC8673869 34814776

